# Non-coding RNAs in necroptosis, pyroptosis, and ferroptosis in cardiovascular diseases

**DOI:** 10.3389/fcvm.2022.909716

**Published:** 2022-08-04

**Authors:** Yuxi Cai, Yiwen Zhou, Zhangwang Li, Panpan Xia, Xinxi ChenFu, Ao Shi, Jing Zhang, Peng Yu

**Affiliations:** ^1^The Second Clinical Medical College of Nanchang University, The Second Affiliated Hospital of Nanchang University, Nanchang, China; ^2^Department of Metabolism and Endocrinology, the Second Affiliated Hospital of Nanchang University, Nanchang, China; ^3^Institute for the Study of Endocrinology and Metabolism in Jiangxi Province, Nanchang, China; ^4^Branch of National Clinical Research Center for Metabolic Diseases, Nanchang, China; ^5^School of Medicine, University of Nicosia, Nicosia, Cyprus; ^6^School of Medicine, St. George University of London, London, United Kingdom; ^7^Department of Anesthesiology, The Second Affiliated Hospital of Nanchang University, Nanchang, China

**Keywords:** non-coding RNAs, necroptosis, pyroptosis, ferroptosis, cardiovascular diseases, immunology

## Abstract

Accumulating evidence has proved that non-coding RNAs (ncRNAs) play a critical role in the genetic programming and gene regulation of cardiovascular diseases (CVDs). Cardiovascular disease morbidity and mortality are rising and have become a primary public health issue that requires immediate resolution through effective intervention. Numerous studies have revealed that new types of cell death, such as pyroptosis, necroptosis, and ferroptosis, play critical cellular roles in CVD progression. It is worth noting that ncRNAs are critical novel regulators of cardiovascular risk factors and cell functions by mediating pyroptosis, necroptosis, and ferroptosis. Thus, ncRNAs can be regarded as promising therapeutic targets for treating and diagnosing cardiovascular diseases. Recently, there has been a surge of interest in the mediation of ncRNAs on three types of cell death in regulating tissue homeostasis and pathophysiological conditions in CVDs. Although our understanding of ncRNAs remains in its infancy, the studies reviewed here may provide important new insights into how ncRNAs interact with CVDs. This review summarizes what is known about the functions of ncRNAs in modulating cell death-associated CVDs and their role in CVDs, as well as their current limitations and future prospects.

## Introduction

Despite significant advances in prevention, diagnosis, and early intervention, cardiovascular diseases (CVDs) remain the leading cause of global mortality and a significant contributor to a decline in quality of life for many people (1, 2). According to the Global Burden of Disease Cooperation Organization's official statistics, CVD patients increased from 271 million in 1990 to 523 million in 2019, nearly doubling in < 30 years. Furthermore, CVD-related deaths have risen steadily from 12.1 million in 1990 to 18.6 million in 2019 (1). CVDs include a wide range of disorders, such as atherosclerosis, ischemia-reperfusion injury, myocardial infarction, uremic cardiomyopathy, cardiac hypertrophy, and others, and are also the leading cause of disability and death worldwide (3).

The pathophysiology of CVDs is complicated (4). Cell death plays a vital role in the pathophysiology of CVDs. Programmed cell death (PCD) is a type of death that is both independent and organized. Apoptosis is widely regarded as the most common mode of cell death and the first type of modulated cell death to be discovered. PCD currently involves pyroptosis, necroptosis, ferroptosis, autophagy, and apoptosis. Cells that have been programmed to die have a significant impact on cardiovascular disease (5). Necroptosis, for example, is activated in advanced atherosclerotic plaques in humans and can be used as a target for experimental treatment and diagnostic intervention in atherosclerosis (6).

ncRNA is a link that cannot be ignored in the occurrence and development of CVDs. ncRNAs account for 98–99% of the human genome and are involved in modulating the expression of genes encoding proteins (7), which can be categorized into long non-coding RNAs (lncRNA), micro RNA (miRNA), circular RNAs (circRNAs), small nuclear RNA (snRNA), piwi-interacting RNA (piRNA), small interfering RNA (siRNA), and small nucleolar RNA (snoRNA) (8). miRNAs are single-stranded RNA molecules about 22 nucleotides in length that can downregulate gene expression at the post-transcriptional level (9). lncRNAs are transcripts of over 200 nucleotides in length that participate in practically every step of gene expression modulation (10). circRNA acts as a novel endogenous closed-chain covalent RNA that is largely expressed in mammalian cells and participate in the transcriptional or post-transcriptional adjustment of gene expression (11). lncRNAs and circRNAs can serve as competing for endogenous RNAs (ceRNAs) to modulate miRNAs, thus interacting with their corresponding mRNA targets. Of note, miRNAs and circRNAs possess the advantage of outstanding stability, resistance to degradation caused by various enzymes, and the ability to circulate in body fluids for an extended duration owing to their short length and circular structure (7). As described in previous studies, small ncRNAs have emerged as critical regulators of gene expression in various cellular pathways and systems (8). One of the most classic action modes of ncRNAs is that miRNAs directly target the partially complementary sites located in the 3′ untranslated region of mRNA and inhibit their expression (12).

Furthermore, ncRNAs play a role in the precise regulation of PCD. In the past few years, an increasing number of studies have identified that ncRNA has functional roles in regulating traditional PCD, such as apoptosis, necrosis, and autophagy (13). These types of cell death can contribute to the progression of certain diseases by regulating the cell cycle through the modulation of their downstream corresponding proteins or genes. In 2022, Xu's study found that overexpression of circ_0007059 can contribute to apoptosis in cardiac tissues of the MI mouse model by regulating its downstream miR-378 and miR-383 (14).

Recently, newly discovered PCDs, such as necroptosis, pyroptosis, and ferroptosis, have been studied, with ample evidence indicating that PCD is a potential key factor in the CVD process (15–17). Lately, ncRNAs have become increasingly crucial in CVDs (coronary heart disease, atherosclerosis, myocardial infarction, and heart failure) (18–21). Through various pathways, ncRNAs regulate gene expression in various cardiovascular diseases. Concurrently, recent research discovered that ncRNAs play an important role in CVDs by modulating PCD.

This review summarizes the progress of the regulatory mechanisms of necroptosis, pyroptosis, and ferroptosis in CVDs and the role of ncRNAs, particularly circRNAs, lncRNAs, and miRNAs, in modulating PCD in CVDs. Identifying these cardiovascular disease-associated ncRNAs will help us understand how CVDs work and provide some new targets and implications for their prevention and treatment. Furthermore, we provide an update on recent developments and perspectives for the clinical and therapeutic use of ncRNAs on pyroptosis, necroptosis, and ferroptosis in CVDs, and their current limitations and prospects for future research.

## Necroptosis in CVDs

### Necroptosis signaling pathway

**Figure 1:**
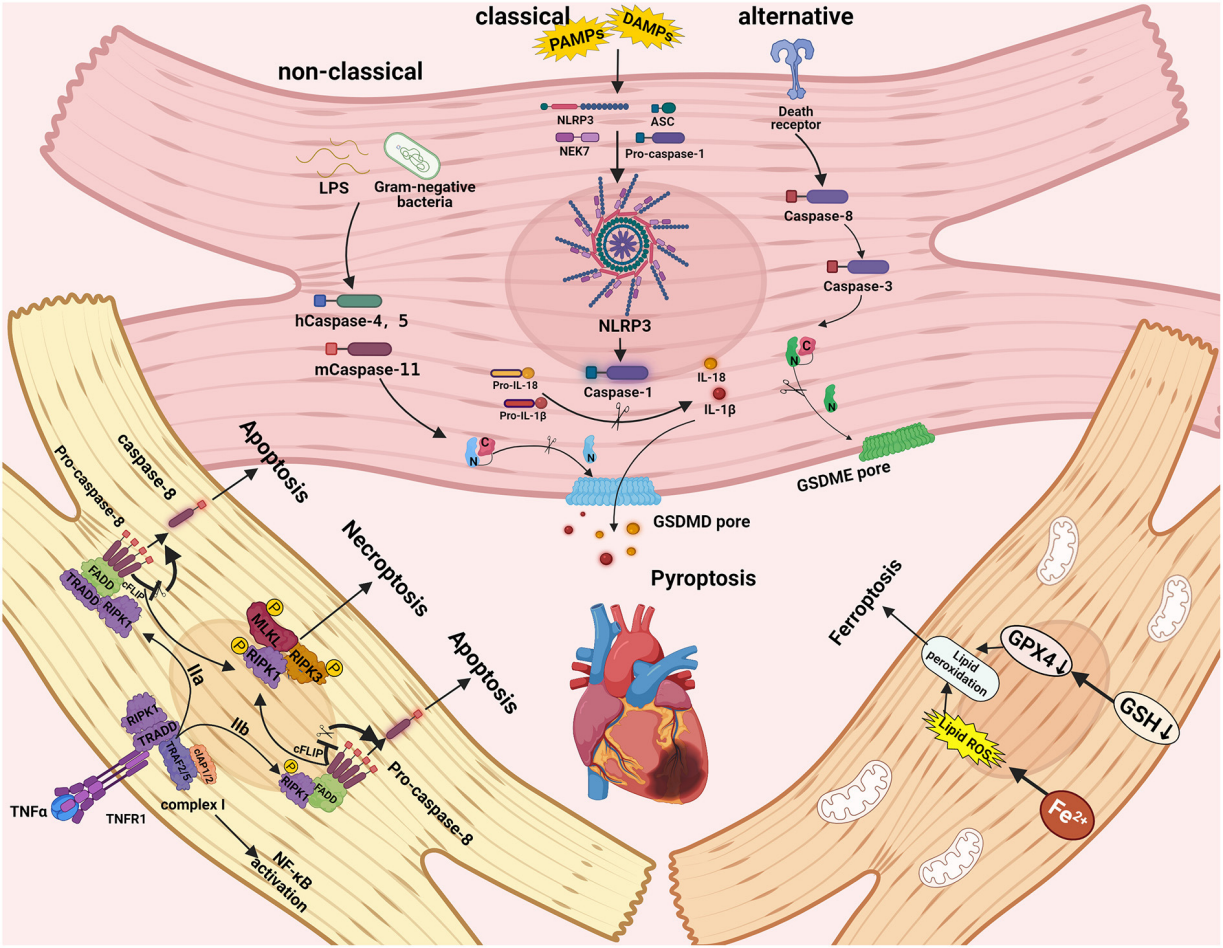
The molecular signaling mechanisms and pathways of pyroptosis, necroptosis, and ferroptosis in cardiac cells. Necroptosis can be triggered by RIPK3 and RIPK1, which recruit MLKL to form the necrosome. The caspase-1 induced classical inflammasome pathway, caspase-4, caspase-5, and caspase-11 induced non-classical pathways, and caspase-3 and caspase-8 induced alternative pathways can trigger pyroptosis. Ferroptosis can be activated by iron accumulation and low levels of GSH-induced lipid peroxidation.

Necroptosis is a type of necrosis controlled by death receptors (22). TNF can initiate necroptosis in the first step by binding to its receptor TNFR1, resulting in the formation of complex I, which includes cIAP1/2, RIPK1, TRAF2/5, and TRADD (23, 24). The ubiquitination of RIPK1 in complex I is mediated by clAP1/2 and TRAF2/5, resulting in complex I stability, which in turn motivates the activation of the classical NF-κB pathway and results in cell survival (25). Meanwhile, cylindromatosis (CYLD) and A20 can deubiquitinate RIP1, leading to the formation of complex IIa, which includes pro-caspase-8, FADD, and TRADD, and can activate RIP1-independent apoptosis (23, 26, 27). When the expressions of cIAP, TGF-activated kinase 1 (TAK1), or NF-κB essential modulator (NEMO) are suppressed, the complex IIb, which includes RIPK1, FADD, and pro-caspase-8, is activated, resulting in RIP1-dependent apoptosis. (28, 29). When the level of FLIP_L_ (FLICE inhibitory protein), which interacts with caspase-8, is high, the catalytic activity of caspase-8 is impaired, resulting in necroptosis (30). RIPK1 and RIPK3 communicate with each other through the receptor homology domain (RHD), which results in the formation of necrosomes and the induction of downstream signaling, ultimately resulting in necroptosis (31). After activation, RIPK3 transports MLKL (a mixed-line kinase-like protein) to the plasma membrane for phosphorylation, resulting in necrosis, changes in cell membrane permeability, and, eventually, cell death (32). The specific molecular signaling mechanism of necroptosis is depicted in Figure 1.

### Contributions of necroptosis to the pathophysiology of CVDs

Necroptosis is involved in the pathogenesis of various CVDs, including AS, I/R injury, and MI.

In the early stage of AS, circulating monocytes enter the subintima to phagocytose ox-LDL through damaged vascular epithelial cells, turning them into foam cells. Subsequently, the expression of RIP3 and MLKL in foam cells is upregulated so that necroptosis is activated, manifesting as inflammation, ultimately boosting the development of AS (33).

When myocardial I/R injury occurs, necroptosis will also arise soon afterward by triggering the RIP1-RIP3-MLKL complex, which can exacerbate myocardial oxidative stress and aggravate I/R injury, simultaneously accompanied by an inflammatory response (34).

RIP3-mediated inflammation is instrumental to the pathogenesis of MI. This inflammatory response results in adverse ventricular remodeling after MI by promoting ROS production (35). Additionally, chemical inhibition of RIP1, another component of the RIP1-RIP3-MLKL complex, can cut down the size of the necrotic myocardium induced by MI (36).

### ncRNAs regulate necroptosis in CVDs

ncRNA manifests as a complete axis that connects the occurrence of PCD with the development of CVDs. In this axis, lncRNA and circRNA compete with target miRNA for the 3′UTR binding site of mRNA to play a negative role in regulating downstream mRNA expression. The activation of this axis can result in the release of tremendous inflammasome that induces the corresponding PCD-like necroptosis and promotes the process of various CVDs. In addition, lncRNA and circRNA can act as “ molecular sponges” to inhibit miRNA expression to participate in the gene expression modulation, thus influencing the development of CVDs.

miRNAs play critical roles in ischemia-reperfusion (I/R) injury (37). I/R injury is defined as a pathological situation characterized by an initial reduction or interruption of blood flow to the organ, followed by restoration of perfusion and reoxygenation, which can result in worsening tissue damage and severe inflammation, as well as severe cell damage and death (38, 39). Qin's team previously demonstrated that miR-223-5p/3p duplex regulated death receptors (DR6 and TNFR1) protein levels and NLRP3 inflammasome signaling pathway, which contributed to the inhibition of myocardial I/R-induced necroptosis (40). Tan et al. discovered that in a mouse model of myocardial I/R injury, RIPK1 was upregulated, worsening myocardial I/R injury *via* the TNF signaling pathway. At the same time, miR-24-3p, which can protect the heart by suppressing RIPK1, was downregulated (41).

Similarly, miRNAs play a role in myocardial infarction (MI) pathology. MI, also known as a heart attack, is most commonly caused by reduced or interrupted blood flow to a part of the heart, resulting in cardiac cell death. In addition, because the epicardial artery is important in feeding the heart muscle, a blood clot embedded in it is usually the primary cause of ischemic myocardium necrosis. However, it is now recognized that blood clot etiology is not required in all cases. The blood supply must match the oxygen demand of all living tissues, including the heart muscle. This is referred to as a supply–demand relationship. In the absence of blood clots, it is now known that if the heart rate is too high or the blood pressure drops, this imbalance in the ratio will damage the heart muscle (42). According to Zhang et al. (43), miR-325-3p overexpression induced by a specific agomiR significantly reduced MI-associated symptoms by suppressing the RIPK3-based necroptosis pathway, indicating that miR-325-3p upregulation could improve MI. Zaafan et al. found that silencing miR-103 inhibited necroptosis and MI induction by targeting fas-associated protein with a death domain (FADD), which was previously shown to be an essential negative regulator of necroptosis (44, 45). Cardiomyocyte progenitor cells (CMPCs) are thought to be a potential source of cell transplantation therapy for promoting myocardial recovery. Furthermore, a recent study found that miR-155 overexpression could reduce necrotic cell death by 40±2.3% in CMPCs by targeting receptor-interacting serine/threonine-protein kinase 1 (RIPK1) (46).

miRNAs play an important role in atherosclerosis (AS). AS is an inflammatory disease of the arterial intima caused by lipids. The final clinical outcome is determined by balancing its pro-inflammatory and anti-inflammatory mechanisms. Macrophages are primarily responsible for the infiltration, modification, and uptake of intimal plasma-derived lipoproteins, resulting in the formation of lipid-filled foam cells and atherosclerotic lesions (47). Recent research found that deleting HIF-1α from myeloid cells could reduce AS and necrotic core formation in apolipoprotein E-deficient mice by inhibiting macrophage necroptosis. By acting on 2, 4-dienoyl-CoA reductase, HIF-1α can mechanically upregulate miR-210, resulting in necroptosis and a reduction in mitochondrial respiration. Simultaneously, HIF-1-mediated downregulation of miR-383 in inflammatory macrophages increased ATP depletion by suppressing poly (ADP-ribose) glycohydrolase, which exacerbated atherosclerosis in lesional macrophages and macrophages derived from inflammatory bone marrow (48).

In other CVDs like myocardial necroptosis prompted by Se deficiency, Yang's study found that overexpression of miR-200a-5p rendered receptor-interacting serine/threonine kinase 3 (RIP3)-dependent necroptosis in cardiomyocytes and animal models by targeting gene ring finger protein 11 (RNF11) (49). Jiang et al. discovered that by targeting MLKL in human HK2 cells, hsa-miR-500a-3P could attenuate toxic and ischemic insults caused by cell necroptosis and the inflammatory response in acute kidney injury (AKI) (50). Furthermore, Huang's research discovered that miR-223-3p was upregulated in AKI caused by 3-chloro-1, 2-propanediol (3-MCPD)-dipalmitate, and regulated RIPK3 expression by targeting the 3′UTR of RIPK3 (51). Similarly, Gu et al. reported in sepsis that miR-425-5p could target the 3′UTR of RIPK1 mRNA to suppress RIPK1 expression and activate RIPK1, reversing the suppression of necroptosis and inflammasome induced by miR-425-5p in septic hepatocytes (52).

There have only been a few reports that lncRNA regulates necroptosis in CVDs. Previous research in I/R injury found that necrosis-related factor (NRF), a long non-coding RNA, regulated cardiomyocyte necrosis by targeting miR-873, which inhibited RIPK1/RIPK3 translation and suppressed RIPK1/RIPK3-mediated necroptosis in cardiomyocytes induced by I/R injury (53).

**Figure 2:**
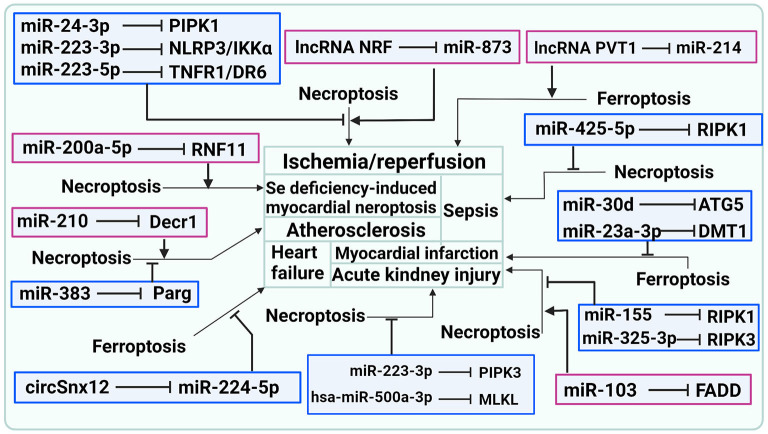
ncRNAs are involved in CVDs by influencing the occurrence of necroptosis and ferroptosis.

In general, what has been discussed thus far indicates that ncRNA plays an important role in CVDs by regulating necroptosis, as shown in Table 1 and Figure 2.

**Table 1:** Non-coding RNAs regulate necroptosis in various type of CVDs.

**Disease**	**ncRNA**	**Experimental phenotype**	**Target gene**	**Repression/Induction of necroptosis**	**References**
**miRNA**					
Ischemia/reperfusion(I/R)	miR-24-3p	C57BL/6 mice cardiomyocytes	RIPK1	Repression	(41)
	miR-223-3p	Pre-miR-223 transgenic (TG) mouse cardiomyocytes	NLRP3/IKKα	Repression	(40)
	miR-223-5p	pre-miR-223-knockout (KO) mouse cardiomyocytes	TNFR1/DR6	Repression	(40)
Atherosclerosis (AS)	miR-210	inflammatory bone marrow-derived macrophages	Decr1	Induction	(48)
	miR-383	inflammatory bone marrow-derived macrophages	poly(ADP-ribose)-glycohydrolase (Parg)	Repression	(48)
Acute kidney injury	miR-223-3p	C57 BL/6 mice tubular cell	RIPK3	Repression	(51)
	has-miR-500a-3p	human tubular epithelial cells	MLKL	Repression	(50)
Se deficiency-induced myocardial necroptosis	miR-200a-5p	Se-deficient chicken cardiomyocytes	RNF11	Induction	(49)
Myocardial infarction (MI)	miR-103	Mice's heart tissue with isoprenaline-induced myocardial infarction	FADD	Induction	(44)
	miR-155	cardiomyocyte progenitor cells	RIPK1	Repression	(46)
	miR-325-3p	C57BL/6 mice cardiomyocytes	RIPK3	Repression	(43)
Sepsis	miR-425-5p	C57BL/6 mice liver cell	RIPK1	Repression	(52)
**lncRNA**					
I/R injury	lncRNA NRF	neonatal mouse cardiomyocytes	miR-873	Induction	(53)

### How to detect necroptosis

The activation of RIPK1, RIPK3, and MLKL is core links in the necroptosis pathway, which are associated with their phosphorylation. Accordingly, phosphorylation of RIP1, RIP3, and MLKL can be used as the hallmarks of the induction of necroptosis *in vivo* and *vitro* (54). It is of note that two newly developed anti-phosphorylated RIP1 antibodies have been explored as biomarkers for the sensitization of RIP1, which respectively recognize the phospho-Ser166 and phospho-Ser14/15 of hRIP1 (55).

## Pyroptosis in CVDs

### Pyroptosis signaling pathway

Pyroptosis, also known as a form of distinct programmed cell death; compared to other forms (e.g., apoptosis and autophagic cell death), its morphology is unique that is influenced by inflammatory caspases (56, 57), as first demonstrated in the death of macrophages caused by Salmonella (58). Classical inflammatory pathways mediated by caspase-1 and non-classical inflammatory pathways mediated by caspase-4/5/11 can both induce pyrolysis (59). Activated caspases can also cleave and multimerize members of the gasdermin family, including GSDMD. During the process of pyroptosis, active GSDMD-NT is transferred to the plasma membrane and forms pores, resulting in water inflow, cellular edema, and dissolution during pyroptosis (60, 61). Pathogen-associated molecular patterns (PAMPs) or endogenous damage-associated molecular patterns (DAMPs) can recognize and combine with PRRs in the non-classical pathway, including NLPR3, the best-studied inflammasomes receptor that plays an important role in immune defense (62). NLPR3 interacts with apoptosis-associated speck-like protein containing a CARD (ASC) *via* the PYD domain after recognizing a signal. ASC is a speck-like protein with an N-terminal PYD domain and a C-terminal CARD domain. The inflammasome is formed by recruiting the CARD domain of ASC, recognizing, and interacting with pro-caspase-1. Caspase-1 activation can result in the cleavage of pro-IL-18 and pro-IL-1β and the release of mature IL-18 and IL-1β (63). While in a non-classical way, gram-negative bacterium's LPS can trigger caspase-11 in mice by binding with their CARD domains, caspase-4 and caspase-5 can also bind with LPS, leading to caspase-4/5/11 oligomerization and excitation in humans (64). Following activation, caspase-4, caspase-5, and caspase-11 cleave GSDMD to generate GSDMD-NT, which can self-assemble in the plasma membrane, causing pores to form and motivating the NLRP3 inflammasome to activate maturation and release of cytokines IL-1β and IL-18, ultimately promoting pyroptosis (65). It is worth noting that alternative pathways mediated by caspase-8 and caspase-3 can also result in pyroptosis. Caspase-8 acts as an apoptotic initiator, while caspase-3 acts as an executioner caspase, cleaving gasdermin E (GSDME) to form an N-terminal fragment of GSDME (GSDME-NT), promoting ATP-induced pyroptosis in macrophages (66). Caspase-8 also causes gasdermin D cleavage, which exacerbates pyroptosis in Yersinia infections (67). The molecular signaling mechanism of pyroptosis is depicted in Figure 1.

### Contributions of pyroptosis to the pathophysiology of CVDs

Pyroptosis plays a critical role in the pathophysiology of different CVDs.

In I/R injury, although ischemia itself can damage the heart muscle, most of the myocardial infarctions stem from injury during reperfusion after a transient period of coronary artery occlusion. The occurrence of pyroptosis results from the activation of caspase and the cleavage of GSDMD induced by inflammasomes. This active fragment creates large pores in the cell membrane to destroy the myocardiocyte, thus aggravating myocardial injury (68).

Vascular endothelial cells (VEC) damage is indispensable to As lesions. The caspase-1 inflammasome pathway can upregulate pyroptosis-related proteins and ultimately trigger VEC pyroptosis, which brings about the loss of endothelium integrity and the increase of vascular permeability, thus promoting As development (69).

The inflammatory response is involved in the development of cardiac hypertrophy (CH) and heart failure (HF). NLRP3 inflammasome components have been discovered in cardiomyocytes, resulting in pyroptosis. This process eventually causes cardiomyocyte proliferation and cytoskeletal remodeling, which can exacerbate the impairment of cardiac function and promote the development of CH and HF (70, 71).

Cardiac fibrosis, one of the primary pathogenesis of diabetic cardiomyopathy (DC), plays a significant role in the process of DC induced by cardiac fibroblasts (CFs) (70). NLRP3 inflammasome components have also been identified in CFs. Hyperglycemia (HG) -induced ROS overproduction contributes to the activation of the NLRP3 inflammasome, of which components have been identified in CFs. In this process, pyroptosis will occur and eventually lead to dysfunction in CFs, thus promoting cardiac fibrosis in DC (72).

### ncRNAs regulate pyroptosis in CVDs

#### miRNAs

MicroRNAs (miRNAs) carry out a variety of cellular functions (73). Recent research has shown that miRNA plays an important role in regulating pyroptosis in CVDs (16, 74).

NLRP3 plays an important role in the development of I/R injury, acting as a molecular platform and activating caspase-1 and cleaving pro-IL-1β, pro-IL-18, and GSDMD (75). A recent study discovered that miR-703 targets NLRP3 and inhibits its expression. Overexpression of miR-703 inhibited the upregulation of NLRP3, caspase-1 mRNA, and protein expression, reducing cardiomyocyte pyroptosis, implying that pyroptosis can be induced *via* the NLRP3/caspase-1 signaling pathway during the I/R-induced cardiomyocyte injury (76). Similarly, miR-135b may inhibit pyroptosis *via* NLRP3/caspase-1/IL-1β pathway, thereby reducing myocardial damage and protecting cardiac function (77). Furthermore, exosomal miR-320b protects the myocardium from I/R damage by inhibiting the production of pyroptosis-related proteins such as NLRP3 and caspase-1 (78).

SIRT1 is another important pyroptosis molecule in I/R injury. SIRT1/PGC-1α/Nrf2 signaling has been shown to mediate oxidative stress, which is important in myocardial I/R injury (79, 80). SIRT1 deficiency was associated with I/R injury. Furthermore, inhibiting miR-29a may improve myocardial I/R injury by downregulating SIRT1, as it can reduce oxidative stress and NLRP3-mediated pyroptosis, thereby protecting I/R injury (81). Similarly, miR 132 can directly target SIRT1 and suppress its expression to prevent oxidative stress and pyroptosis in myocardial cells (82).

Furthermore, FOXO3 is a key player in I/R injury, which increases oxidative damage and decreases myocardial function (83). A previous study found that a high level of miR-100-5p in hucMSC-exo could inhibit FOXO3 expression while suppressing the activation of NLRP3 inflammasomes, limiting cytokine release and protecting against I/R-induced pyroptosis (84). Yao et al. discovered that miRNA-1 could mediate the downregulation of PIK3R1 expression while upregulating FOXO3 and caspase-1 expression as well as the production of IL-1β and IL-18, which induced pyroptosis in cardiomyocytes injured by I/R (85). Then, by targeting FOXO3, miR-149 inhibition could also downregulate inflammatory factors like IL-18 and IL-1β in both mRNA and protein levels, preventing cardiomyocyte pyroptosis (86). FOXO3a, on the other hand, is a positive regulator of pyroptosis. miR-29b acted as a negative regulator, inhibiting the protective effect of FOXO3a and its downstream protein ARC, causing pyroptosis in I/R-injured myocardial cells *via* FOXO3a/ARC axis (87).

Other molecules, such as CRISPLD2, RP105, and MCL-1, also play protective roles in cardiomyocyte pyroptosis. By directly targeting CRISPLD2 and regulating cardiomyocyte pyroptosis, miR-424 promoted cardiac I/R injury (88). Piperine protected against pyroptosis in myocardial I/R injury by mediating the miR-383/RP105/AKT pathway, and RP105 worked as a miR-383 target (89). Exosomal miR-29a from macrophages treated with I/R plays a role in mediating cardiomyocyte pyroptosis by targeting MCL-1 (90). TXNIP, on the other hand, acts as a positive regulator in pyroptosis. M2-exos increased miR-148a expression, which could mediate TXNIP downregulation and inactivation of the TLR4/NF-κB/NLRP3 inflammasome signaling pathway to prevent myocardial pyroptosis caused by I/R injury (91).

In AS, miR-125a-5p mediated oxLDL-induced pyroptosis in vascular endothelial cells (VECs) by downregulating TET2 and increasing NF-κB activation, which activated NLRP3 and caspase-1, contributing to VECs and AS pyroptosis (92). TET2, as a DNA demethylase, protects VECs from the dysfunction caused by oxLDL in hydrogen sulfide (93). Furthermore, miR-125a-5p inhibition reduced ROS production by improving DNA methylation and mitochondrial function, as well as downregulating the expression of pyroptosis-related proteins, such as IL-1β, caspase-1, and NLRP3 (93). Peng et al. discovered that miR-30c-5p could inhibit NLRP3 inflammasome-mediated endothelial cell pyroptosis in AS by downregulating FOXO3 expression (94). In addition to oxLDL, shear stress played an important role in the onset and progression of AS, causing dramatic changes in signaling and gene expression in endothelial cells (ECs), regulating cell migration, proliferation, growth, and death (95). With the augmented expression of inflammasome-dependent pyroptosis in human umbilical vein endothelial cells (HUVECs), shear stress suppressed mechanosensitive miR-181b-5p expression, and miR-181-5p also inhibited signal transducer and transcriptional activation factor 3 (STAT3) gene expression by directly binding to its 3′UTR, leading to the downregulation of NLRP3 transcription and NLRP3 inflammasome-related pyroptosis (96). Notably, piceatannol inhibits pyroptosis in macrophages by upregulating Nrf2, which is known for its antioxidant and anti-inflammatory properties (97), *via* the miR-200a/Nrf2/GSDMD axis (81). Furthermore, miR-200a suppressed Nrf2 expression, resulting in increased levels of IL-18, IL-1β, and caspase-1.

miRNAs play an important role in cardiac hypertrophy (CH) and heart failure (HF). CH causes changes in cardiomyocytes such as calcium processing, metabolism, and gene expression, as well as cell death (apoptosis and autophagy), extracellular matrix (ECM) changes (fibrosis), and angiogenesis. HF is a crippling condition in which the heart fails to supply oxygen to the body. The heart initially responds to additional stress or heart damage in a compensatory manner, increasing its volume and mass to normalize wall stress and cardiovascular function at rest. Pathological CH refers to the typical enlargement of the heart (98). According to a recent study, MLK3 can promote pyroptosis of cardiomyocytes primarily *via* NFκB/NLRP3 signaling pathway, aggravating myocardial fibrosis in the early stages of chronic heart failure (CHF). The cardiac function of mice subjected to transverse aortic constriction (TAC) could then be significantly improved due to MLK3′s suppressive effect *via* upregulation of miR-351 expression (99). Similarly, Zhu et al. discovered that targeting IKKε to suppress pyroptosis could reduce cardiomyocyte hypertrophy caused by Ang II (100).

Furthermore, Fan et al. discovered that miR-599 inhibited pyroptosis caused by H_2_O_2_ by downregulating the expression of ASC as well as the downstream inflammatory factors IL-18 and IL-1β in oxidative stress-induced cardiac injury (101). Another group reported that overexpression of the lncRNA TUG1 alleviated NLRP3-dependent pyroptosis of cardiomyocytes by regulating miR-186-5p/XIAP axis in a study on coronary microembolization (CME)-induced myocardial damage.

Diabetic cardiomyopathy (DC), another common type of CVD, is defined as cardiac dysfunction in diabetic patients who do not have other cardiovascular diseases (102). Inflammation, oxidative stress, impaired immune regulation, improper activation of the renin-angiotensin-aldosterone system, abnormal subcellular components, and systemic metabolic disorders all contribute to heart stiffness/diastolic dysfunction, interstitial fibrosis of cardiac tissue, and posterior systolic dysfunction in diabetic cardiomyopathy (103). According to Jeyabal et al., miR-9 inhibition increased ELAV-like protein 1 (ELAVL1) expression as well as IL-1β and caspase-1 expression, whereas miR-9 mimic transfection decreased caspase-1, IL-1β, and ELAVL1 expression, which inhibited cardiomyocyte pyroptosis (104). Furthermore, upregulation of miR-30d in streptozotocin (STZ)-induced diabetic rats and high-glucose-treated cardiomyocytes aggravated pyroptosis by decreasing the concentrations of FOXO3a as well as its downstream protein like ARC and resulted in high expression of pro-pyroptosis factors or cytokines like IL-18, IL-1β, and caspase-1 (105). Furthermore, in STZ-induced diabetic cardiac fibrosis, miR-21–3p exacerbates pyroptosis of cardiomyocytes by suppressing AR expression *via* the NLRP3/caspase-1 pathways (106). miRNAs are also important in other CVDs. Sepsis-induced myocardial dysfunction (SIMD) is now thought to be the inherent systolic and diastolic myocardial dysfunction on the left and right sides of the heart caused by sepsis. (107, 108).

Recent research in SIMD found that the lncRNA ZFAS1, which is activated by the transcription factor SP1, reduces the expression of miR-590-3p, which regulates the AMPK/mTOR signal pathway, influencing NLRP3-dependent pyroptosis of cardiomyocytes and promoting SIMD (109). miRNAs also played a role in the pathogenesis of uremic cardiomyopathy (UC), another fatal cardiac disease. Diastolic insufficiency is a common feature of UC in patients with chronic kidney disease (CKD) who also have left ventricular hypertrophy (LVH) and myocardial fibrosis (110). Wang et al. discovered that macrophage-derived miR-155–containing exosomes aggravated pyroptosis and alterations (CH and cardiac tissue fibrosis) in UC by directly targeting FOXO3a (111). miRNAs also play important cellular roles in aortic dissection (AD). AD is a potentially fatal disease caused by the rupture of the aortic intima or bleeding of the aortic wall, which results in aortic wall detachment (112). Macrophages have dual anti-inflammatory and anti-inflammatory functions, play a critical role in aortic wall inflammation, and contribute to the development of AD and AD complications (113–116). A recent study found that miR-133a could negatively regulate the expression level of NLRP3 by specifically binding to its 3′UTR and that upregulating miR-133a inhibited pyroptosis induced by LPS in vSMCs (117). Similarly, in coronary artery disease (CAD), Wang et al. discovered that upregulation of miR-223 significantly reduced oxLDL-induced cell death, effectively eliminating NLRP3 inflammasome-mediated pyroptosis in human VECs (118).

#### lncRNAs

Similar to miRNAs, lncRNAs function in the regulation of pyroptosis in CVDs (119). In MI, lncRNA KLF3-AS1 in hMSC exosomes acted as a ceRNA to sponge miR-138-5p, which targeted SIRT1. Interfering with miR-138-5p increased the KLF3-AS1 expression while decreasing the level of IL-18, IL-1β, cleaved-caspase-1, ASC, cleaved-GSDMD, and NLRP3, indicating that NLRP3-mediated pyroptosis occurred *via* the miR-138-5p/SIRT1 axis in MI (120). Han et al. reported that overexpression of CYP1B1 abolished the suppressive effect of lncRNA H19 on pyroptosis, whereas H19 inhibited the activity of CYP1B1 promoters by targeting PBX3 to inhibit IL-18, IL-1β, ASC, and NLRP3 in hypoxic cardiomyocytes (121).

Furthermore, in AS, intragastric administration of melatonin reduced the expression of pyroptosis-related genes, such as cleaved-caspase-1, ASC, NLRP3, GSDMD-N termini, NF-κB/GSDMD cleaved-caspase-1, IL-1β, and IL-18, which significantly reduced atherosclerotic plaques in mouse aortas fed a high-fat diet *via* downregulating lncRNA MEG3, high expression of which prompted pyroptosis in HAECs (122). Song et al. (123) discovered that the lncRNA MALAT1 sponged miR-22 to promote NLRP3 inflammasome expression, causing pyroptosis in human ECs exposed to high glucose. Similarly, as explained in Han's study, pyroptosis was suppressed in HG-oxLDL-treated macrophages due to MALAT1 overexpression *via* the miR-23c/ELAVL1 axis (124). Anti-pyroptosis lncRNANEXN-AS1 was found to be upregulated in atorvastatin-treated HVECs. High lncRNANEXN-AS1 expression reduces the expression of pyroptosis-related genes (IL-18, IL-1β, GSDMD, caspase-1, and NLRP3) by upregulating NEXN (125).

Yang et al. also reported in DC that lncRNA sponged miR-214-3p by competing for binding sites and regulating gene expression. Silencing the lncRNA Kcnq1ot1 reduced pyroptosis and fibrosis in diabetic cardiomyopathy by targeting miR-214, which influenced the expression level of its downstream proteins (e.g., caspase-1, TGF-β1) (126, 127). Xu discovered that overexpression of the lncRNA GAS5 repressed miR-34b-3p expression while increasing aryl hydrocarbon receptor (AHR) expression, which inhibited NLRP3-dependent pyroptosis in DC by playing a role in miR-34b-3p/AHR signaling (128). In addition, in CME-induced myocardial damage, overexpression of lncRNA TUG1 alleviated pyroptosis of cardiomyocytes caused by the NLRP3 inflammasome *via* the miR-186-5p/XIAP axis (129).

#### circRNAs

So far, only a few circRNAs have been proven to play a critical role in CVDs. Yang et al. discovered in DC that the caspase-1-associated circRNA (CACR), hsa_circ_0076631, functioned as a ceRNA, sequestering miR-214-3p and thus alleviating its suppressive effect on caspase-1 expression. Pyroptosis in cardiomyocytes treated with high glucose could thus be significantly reduced (130).

**Figure 3:**
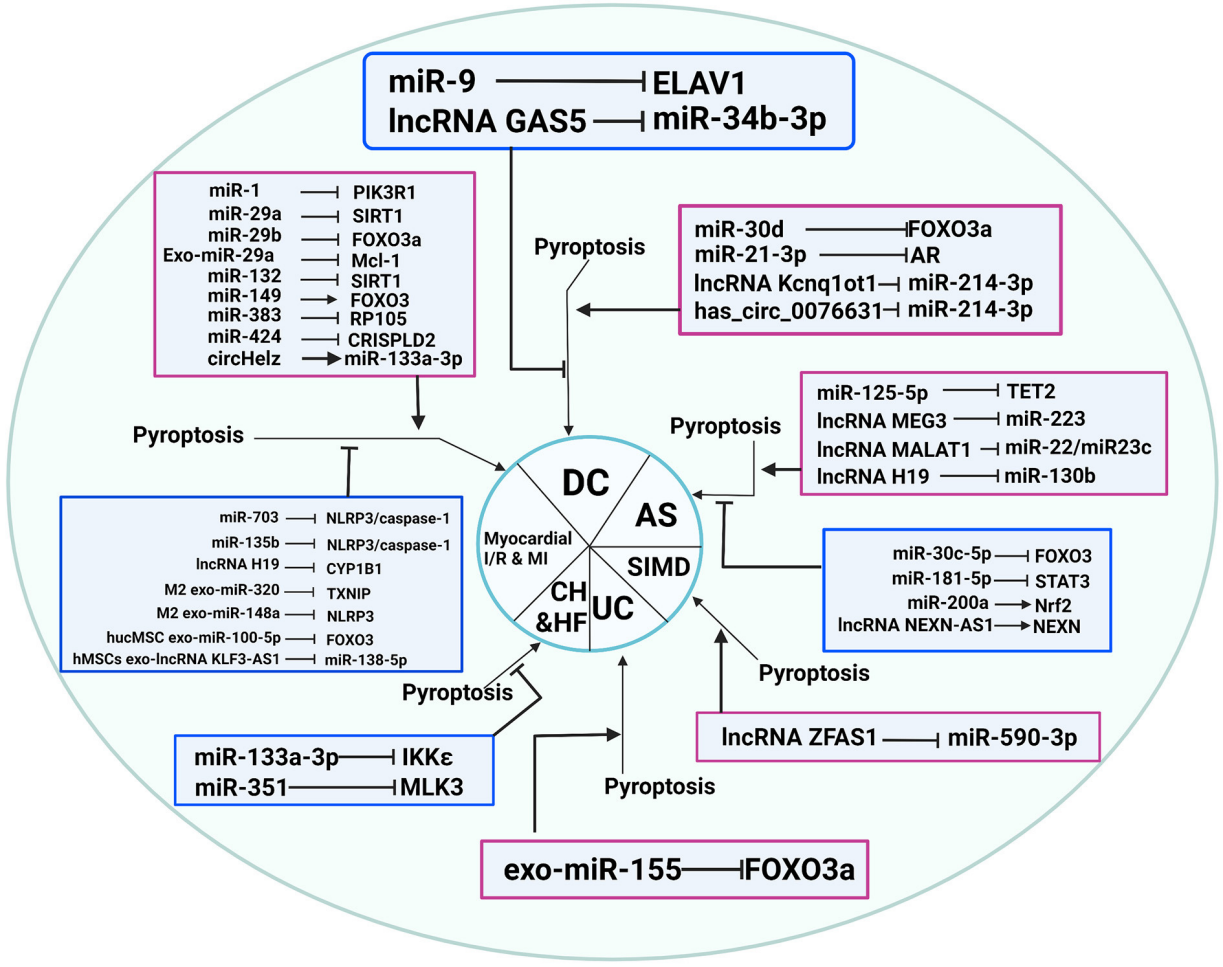
Non-coding RNAs play an essential role in CVDs through pyroptosis regulation. AS, atherosclerosis; CH, cardiac hypertrophy; DC, diabetic cardiomyopathy; HF, heart failure; I/R, ischemia-reperfusion; SIMD, sepsis-induced myocardial dysfunction; UC: uremic cardiomyopathy.

In summary, ncRNAs are demonstrated to play an important role in CVDs through the regulation of pyroptosis, as shown in Table 2 and Figure 3.

**Table 2:** Non-coding RNAs regulate pyroptosis in various types of CVDs.

**Disease**	**ncRNA**	**Experimental phenotype**	**Target gene**	**Repression/** **Induction of pyroptosis**	**References**
**miRNA**					
I/R injury and MI	miR-1	H9c2 myocardial cells	PIK3R1	Induction	(85)
	miR-29a	H9c2 myocardial cells	SIRT1	Induction	(81)
	miR-29b	neonatal rat cardiomyocytes	FoxO3a	Induction	(87)
	Exo-miR-29a	neonatal mouse cardiomyocytes	Mcl-1	Induction	(90)
	hucMSC exo-miR-100-5p	AC16 cells	FoxO3	Repression	(84)
	miR-132	H9c2 myocardial cells	SIRT1	Induction	(82)
	miR-135b	neonatal mice ventricular cardiomyocytes	NLRP3/caspase-1	Repression	(77)
	M2 exo-miR-148a	neonatal rat cardiomyocytes	TXNIP	Repression	(91)
	miR-149	H9c2 myocardial cells	FoxO3	Induction	(86)
	M2 exo-miR-320	neonatal rat cardiomyocytes	NLPR3	Repression	(78)
	miR-383	rat cardiomyocyte	RP105	Induction	(89)
	miR-424	H9c2 myocardial cells	CRISPLD2	Induction	(88)
	miR-703	mouse cardiomyocytes	NLRP3/caspase-1	Repression	(76)
Atherosclerosis	miR-30c-5p	human aortic endothelial cells	FoxO3	Repression	(94)
	miR-125-5p	vascular endothelial cells	TET2	Induction	(92)
	miR-181-5p	human umbilical vein endothelial cells	STAT3	Repression	(96)
	miR-200a	RAW264.7 cells	Nrf2	Repression	(97)
Diabetic cardiomyopathy	miR-9	human ventricular cardiomyocytes	ELAV1	Repression	(104)
	miR-21-3p	neonatal rat cardiacfibroblasts	AR	Induction	(106)
	miR-30d	neonatal rat cardiomyocyte	FoxO3a	Induction	(105)
Uremic cardiomyopathy	exo-miR-155	C57BL/6 cardiomyocytes	FoxO3a	Induction	(111)
Cardiac hypertrophy and heart failure	miR-133a-3p	human cardiomyocyte	IKKε	Repression	(100)
	miR-351	TAC mice cardiomyocyte	MLK3	Repression	(99)
**lncRNA**					
I/R injury and MI	hMSCs exo-lncRNA KLF3-AS1	H9c2 myocardial cells	miR-138-5p	Repression	(120)
	lncRNA H19	H9c2 myocardial cells	CYP1B1	Repression	(121)
Diabetic cardiomyopathy	lncRNA Kcnq1ot1	Cardiac fibroblasts	miR-214-3p	Induction	(126, 127)
	lncRNA GAS5	HL-1	miR-34b-3p	Repression	(128)
Sepsis-induced cardiac dysfunction	lncRNA ZFAS1	neonatal mice cardiomyocytes	miR-590-3p	Induction	(109)
Atherosclerosis	lncRNA MEG3	human aortic endothelial cells	miR-223	Induction	(122)
	lncRNA MALAT1	EA.hy926 cells	miR-22	Induction	(123)
	lncRNA MALAT1	bone-marrow-derived macrophages	miR-23c	Induction	(124)
	lncRNA NEXN-AS1	human vascular endothelial cells	NEXN	Repression	(125)
	lncRNA H19	Raw264.7 cells	miR-130b	Induction	(131)
**circRNA**					
MI	circHelz	neonatal mouse ventricular cardiomyocytes	miR-133a-3p	Induction	(132)
Diabetic cardiomyopathy	hsa_circ_0076631	AC16 cells	miR-214-3p	Induction	(130)

### How to detect pyroptosis

The mechanism of pyroptosis involves three major signaling pathways, all activating downstream GSDMD and E, which leads to the release of IL-1 and IL-18. Accordingly, corresponding markers can be employed for detecting pyroptosis, such as the cleavage of GSDM D and E, release of IL-1β and −18, or caspase-1, −3, −4, −5, −8, and −11. Immunological techniques like western blotting can be applied as the most effective methods to detect or monitor pyroptosis owing to little involvement in transcription and translation during the modulation of pyroptosis (133).

## Ferroptosis in CVDs

### Ferroptosis signaling pathway

Ferroptosis is an oxidation iron-dependent cell death that differs from apoptosis, necroptosis, pyroptosis, and other types of cell death (134). Mechanically, low-glutathione levels induce the downregulation of glutathione peroxidase 4 (GPX4) and intracellular iron (mostly Fe^2+^) accumulation, which improves the level of lipid-based ROS, which activates the high level of expression of unsaturated fatty acids on the cell membrane, ultimately resulting in liposome peroxidation and cell death (135). The ferroptosis pathway involves the ferroptosis induced by inhibition of the cystine-glutamate transport receptor (systemXc-), ferroptosis induced by P53, and ferroptosis driven by GPX4 (136). Regardless of the different upstream pathways, the final effects all lead to the downregulation of glutathione peroxidase (GPXs), which reduces cells' antioxidant capacity and promotes the reaction of lipid peroxidation, resulting in ferroptosis (137). Ferroptosis inducers possess two types: one contains sulfoximine, sulfasalazine, erastin, etc. This kind of inducer suppresses the function of system Xc- and decreases the level of glutathione (GSH), which results in cell redox imbalance; another kind of inducer contains DPI13, RSL3, DPI7, DPI12, DPI10, etc. Synthetic compounds can suppress GPX4 and increase the level of peroxide in cardiomyocytes. Finally, due to the unnatural metabolism of iron ions in cardiomyocytes, the high level of ROS results in ferroptosis (138). The specific molecular signaling mechanism of ferroptosis is demonstrated in Figure 1.

### Contributions of ferroptosis to the pathophysiology of CVDs

Ferroptosis is also considered a crucial part of the pathophysiology of diverse CVDs.

During myocardial I/R injury, large amounts of degraded ferritin lead to the release of tremendous amount of iron into the coronary arteries. Subsequently, excessive iron in coronary arteries can impair cardiac function and exacerbate myocardial damage (139, 140).

In the early and middle stages of MI, the expression of Gpx4 is inhibited, but the content of ROS increases, thus causing the ferroptosis of myocardial cells and the deterioration of cardiac function (141).

Iron homeostasis plays an indispensable role in maintaining myocardial function. Either iron deficiency or iron overload can worsen cardiac function in HF (142).

### ncRNAs regulate ferroptosis in CVDs

Song et al. reported in MI that exosomes derived from HUCB-MSCs could carry miR-23a-3p to protect cardiomyocytes against I/R-induced ferroptosis by downregulating divalent metal transporter 1 (DMT1) expression, thereby attenuating myocardial injury in AMI mice (143). Another relevant study found that the expression of miR-30d decreased in cardiomyocytes after MI, which could inhibit autophagy by binding to autophagy-related protein 5 (ATG5). Furthermore, autophagy after MI might also promote ferroptosis (144).

miR-214 has been reported to mediate iron translocation into cells in I/R injury by modulating transferrin receptor 1 protein (TFR1) *via* binding to 3′UTR of TFR1. Furthermore, overexpression of PVT1 or silencing of miR-214 significantly reduced the effects of Fer-1 on ferroptosis *in vitro* (145).

Zheng et al. discovered in HF that miR-224-5p could bind to the 3′UTR region of ferritin heavy chain 1 (FTH1) and functioned as a downstream target of circSnx12, which regulated miR-224-5p expression. Furthermore, silencing circSnx12 or overexpression of miR-224-5p could cause cardiac cell death by lowering FTH1 expression and directly regulating iron overload in cardiomyocytes (146).

As indicated above, ncRNAs are involved in CVDs by influencing the occurrence of ferroptosis, as shown in Table 3 and Figure 2.

**Table 3:** Non-coding RNAs regulate ferroptosis in various types of CVDs.

**Disease**	**ncRNA**	**Experimental phenotype**	**Target gene**	**Repression/Induction of ferroptosis**	**References**
**miRNA**					
Myocardial infarction (MI)	miR-23a-3p	neonatal mouse ventricular cardiomyocytes	DMT1	Repression	(143)
	miR-30d	H9c2 myocardial cells	ATG5	Repression	(144)
**lncRNA**					
Brain ischemia/reperfusion (I/R)	lncRNA PVT1	C57BL/6 mice	miR-214	Induction	(145)
**circRNA**					
Heart failure	circSnx12	C57/BL6J mice cardiomyocytes	miR-224-5p	Repression	(146)

### How to detect ferroptosis

Ferroptosis is driven by the lethal accumulation of lipid peroxides in plasma membranes. Therefore, the determination of lipid peroxide level plays a key role in analyzing ferroptosis in biological samples. Recently, a newly discovered technique using BODIPY-C11 probe and flow cytometry to detect ferroptosis has been developed, which can assay cellular lipid peroxide levels in live cells with hypersensitivity and high accuracy (147).

## ncRNAs as therapeutic targets in CVDs

Many ncRNAs have been identified as drug targets for treating CVDs in recent years, indicating the great potential of using ncRNAs as therapeutic targets in CVDs by influencing the manner of cell death. Recently, we found that piperine treatment can attenuate pyroptosis *via* downregulating the expression of miR-383 in the rat I/R injury model (148). In the rat model of MI, for example, the β blocker propranolol can reduce miR-1 expression by inhibiting the beta-adrenoceptor–cAMP–protein kinase A (PKA) signaling pathway and suppressing the expression of transcriptional factor serum response factor (SRF) (149), thus the propranolol may improve MI *via* downregulating miR-1 then inhibiting pyroptosis in cardiomyocytes (85). Another study found that Tanshinone IIA, an active component of Chinese medicine *Salvia miltiorrhiza*, could suppress the level of miR-1 in rats 3 months after MI (150). Likewise, in Laura's study, she discovered that Trimethylamine n-Oxide (TMAO), which is metabolized from Choline, L-carnitine, and betaine, can upregulate miR-30c-5p in HEPG-2, THP-1, mouse liver organoids, and primary human macrophages (151). Then, miR-30c-5p is associated with pyroptosis repression, which can lead to AS (94). Schisandrin A has been studied for its protective role in chronic heart failure *via* the upregulation of miR-155, which represses the necroptosis of cardiomyocytes (152). Moreover, we found that nicorandil combined with trimetazidine treatment can attenuate the progress of CHD *via* upregulating the miR-223-3p (153). Additionally, a study found that anti-miR132 can potentially play a role in heart failure treatment, which induces pyroptosis (148). In Wang's study, MIR-17-5P silencing improves myocardial tissue microcirculation by reducing the rate of apoptosis and repairing vascular damage, thereby protecting cardiac function after acute myocardial infarction (154). Moreover, prostaglandin E1 protects cardiomyocytes against hypoxia-reperfusion-induced injury *via* the miR-21-5p/FASLG axis (155). As a result, the studies described above demonstrate the infinite possibilities of targeting ncRNAs for treating CVDs, and more relevant studies will be published soon, contributing to the development of CVD targeted therapy.

## Current limitations and future prospects

### Clinical implication and limitation of ncRNAs in necroptosis, pyroptosis, and ferroptosis of CVDs

Significant progress has been made in the field of ncRNA over the last few decades, particularly in terms of its nature, structure, and function. Part of the research findings has recently been successfully implemented in clinical applications. Except as a therapeutic target, one of its most useful applications in CVDs is as a biomarker. For instance, a study verified that lncRNA MALAT1 could serve as biomarkers with independent predictive properties for diagnosing, severity, and prognosis of patients with sepsis (156). Because biomarkers are an essential tool for CVD screening, diagnosis, and prognosis, using good warning indicators can help doctors identify high-risk cardiovascular diseases as soon as possible, which is important for lowering death rates and improving prognosis. As a result, their clinical application is steadily increasing. Currently, the proliferation of biomedical technologies has broadened the range of new blood-derived biomarkers (157). Many researchers have affirmed that numerous circulating miRNAs are significantly upregulated or downregulated in various CVDs (158, 159). Recent research showed that overexpression of miRNA-195, miRNA-23a, miRNA-24, miRNA-23b, and other miRNAs could lead to cardiomyocyte hypertrophy *in vitro* (159).

When compared to traditional biomarkers, ncRNAs have four distinct advantages as follows: (1) abnormal ncRNA expression is linked to the onset and progression of diseases. (2) ncRNA expression is found in cells, tissues, and organs and has high sensitivity and specificity for diagnosing certain diseases. (3) Furthermore, due to their ability to secrete into blood compartments and other body fluids, ncRNAs are simple to acquire and detect. (4) Finally, ncRNAs are relatively stable and capable of transmitting signals locally or over long distances due to exosome transport (157, 160). Therefore, an increasing body of evidence supports the key role of ncRNA in the pathologic mechanism and development of CVDs. Accordingly, regardless of clinical or preclinical studies, both of them are striving to evaluate the diagnostic and prognostic potency of ncRNA and appraise the effectiveness of the treatment based on ncRNAs for CVDs. For instance, the usual biochemical markers for AMI include MB, CK-MB, cTnI, and cTnT (161). However, their clinical application effect in the early detection of AMI is not ideal and satisfactory. According to Pan et al., the concentrations of cTnI and CK-MB in the AMI group were significantly higher than in the control group, peaking 12 h after the disease onset. The expression level of miR-130 was also strongly upregulated in the AMI class, reaching a peak 6 h after the occurrence of AMI in the AMI class, compared to the administer classification. Circulating miR-130 levels peaked earlier than cTnI and CK-MB, implying that miR-130 levels can improve the diagnostic accuracy of cTnI and CK-MB and provide useful clues for the early detection of AMI (162).

However, several huge challenges must be overcome before the clinical applications of ncRNAs in necroptosis, pyroptosis, and ferroptosis of CVDs: (1) low expression in body fluids: First, the relative abundance of specific lncRNAs may pose a problem. Because, despite the fact that ncRNAs are present in both peripheral blood and whole blood samples, most peripheral blood miRNAs may originate primarily from well-vascularized tissues (163). Low expression of ncRNAs in body fluids makes it difficult to detect their specific biological functions (164). Thus, more powerful techniques are urgently needed to identify ncRNAs that modulate the necroptosis, pyroptosis, and ferroptosis signaling pathways and uncover their exact mechanisms. (2) maintaining the effectiveness and stability: Indeed, it is unclear whether the link between lncRNAs and miRNAs can serve as a potentially powerful therapeutic target. Because low-level lncRNAs bind to highly expressed miRNAs may not cause significant effects (165). In addition, maintaining the stability of ncRNAs is still a major difficulty (166). (3) The complexity of the regulatory mechanism: Additionally, ncRNAs have complex functions in necroptosis, pyroptosis, and ferroptosis of CVDs, influencing a wide range of pathways and biological functions, revealing the unexpected complexity of the clinical applications of ncRNAs in necroptosis, pyroptosis, and ferroptosis of CVDs. Therefore, there is an urgent need to comprehensively identify the distinct pathways, and these functional mechanisms and their applications are waiting for further research. (4) Plasma storage conditions and sample handling: Some researchers hypothesized that long-term storage and improper sample processing could affect the level of some miRNA in plasma, thereby influencing diagnostic results (167–169). (5) The high cost: Finally, the cost may be another considerable blockage since the manufacturing process of ncRNAs is relatively complicated, like controlling RNase activity, reverse transcription-polymerase (PCR), RNA purification, etc. (167, 170).

### Future prospects of ncRNAs in necroptosis, pyroptosis, and ferroptosis of CVDs

In recent years, with the rapid development of quantification and standardization methodologies, the prevalence of ncRNAs as biomarkers has increased due to their disease specificity, accessibility, stability, and other distinguishing features, making ncRNAs valuable as biomarkers (171, 172). Considering the close link between ncRNAs and necroptosis, pyroptosis, and ferroptosis, it holds promise in developing ncRNA-based methods to monitor and interfere with necroptosis, pyroptosis, and ferroptosis in CVDs. Furthermore, the tissue-specific expression patterns of ncRNAs provide a unique possibility to regulate tissue-specific necroptosis, pyroptosis, and ferroptosis in CVDs. With more in-depth research, more necroptosis, pyroptosis, and ferroptosis-related ncRNAs will be discovered, and their underlying mechanisms of the necroptosis, pyroptosis, and ferroptosis pathways will increasingly be deciphered as well. But the fact is that methodological and biological characteristics currently limit the high specificity and sensitivity of ncRNA detection. (e.g., a short sequence of miRNAs, susceptibility to degradation, and kinetics) (173, 174). Consequently, further research is urgently needed to elucidate the relationship between the expression levels of ncRNAs in plasma and tissues. In addition, the quantities, storage conditions, sample handling, and kinetics of circulating ncRNAs should be improved, and the cost of ncRNAs should be cut down. Therefore, altering ncRNAs *in vivo* by novel therapeutics holds promise in developing a novel therapeutic approach to diagnosing and treating necroptosis, pyroptosis, and ferroptosis-related CVDs. Further investigations will continue to strive for these. The future trend and development direction in this field will construct a novel ncRNAs detection strategy with higher sensitivity and specificity (175).

## Conclusion

CVDs are the leading cause of death from nearly all diseases worldwide. Cell death has long been recognized as necessary for our tissues and bodies to maintain their typical morphological character and physiological function, but it has recently been identified as the primary cause of clinical diseases and severe pathological injuries. Recently, it has been reported that new types of PCD, such as pyroptosis, necroptosis, and ferroptosis, play a different role in the progression of CVDs. In this review, we summarized and analyzed the pathway of ncRNAs involved in necroptosis, pyroptosis, and ferroptosis and their impact on CVD pathogenesis, as shown in Figures 2, 3. Thus, revealing the role of ncRNA-regulated programmed cell death in CVDs will identify novel diagnostic markers and potential therapeutic targets in the clinical treatment of CVDs.

## Author contributions

PY and JZ: conceptualization, methodology, funding acquisition, and project administration. YC and YZ: writing original draft and formal analysis. ZL: visualization and supervision. PX: software and investigation. AS and XC: data curation, validation, writing—review and editing and resources. All authors contributed to the article and approved the submitted version.

## Funding

This study was supported by the Natural Science Foundation in Jiangxi Province, China (Grant Nos. 202002BAB216022, 20192ACBL21037, and 202004BCJL23049), the National Natural Science Foundation of China (Nos. 82160371 and 82100869), and National Clinical Research Center for Geriatrics-JiangXi Branch Center (2021ZDG02001).

## Conflict of interest

The authors declare that the research was conducted in the absence of any commercial or financial relationships that could be construed as a potential conflict of interest.

## Publisher's note

All claims expressed in this article are solely those of the authors and do not necessarily represent those of their affiliated organizations, or those of the publisher, the editors and the reviewers. Any product that may be evaluated in this article, or claim that may be made by its manufacturer, is not guaranteed or endorsed by the publisher.
